# Determining cantilever stiffness from thermal noise

**DOI:** 10.3762/bjnano.4.23

**Published:** 2013-03-28

**Authors:** Jannis Lübbe, Matthias Temmen, Philipp Rahe, Angelika Kühnle, Michael Reichling

**Affiliations:** 1 Fachbereich Physik, Universität Osnabrück, Barbarastraße 7, 49076 Osnabrück, Germany; 2 Institut für Physikalische Chemie, Johannes Gutenberg-Universität Mainz, Duesbergweg 10-14, 55099 Mainz, Germany; 3 now at: Department of Physics and Astronomy, The University of Utah, 115 South 1400 East, Salt Lake City, UT 84112, USA

**Keywords:** AFM, cantilever, noncontact atomic force microscopy (NC-AFM), Q-factor, thermal excitation, resonance, spectral analysis, stiffness

## Abstract

We critically discuss the extraction of intrinsic cantilever properties, namely eigenfrequency *f_n_*, quality factor *Q_n_* and specifically the stiffness *k_n_* of the *n*th cantilever oscillation mode from thermal noise by an analysis of the power spectral density of displacement fluctuations of the cantilever in contact with a thermal bath. The practical applicability of this approach is demonstrated for several cantilevers with eigenfrequencies ranging from 50 kHz to 2 MHz. As such an analysis requires a sophisticated spectral analysis, we introduce a new method to determine *k_n_* from a spectral analysis of the demodulated oscillation signal of the excited cantilever that can be performed in the frequency range of 10 Hz to 1 kHz regardless of the eigenfrequency of the cantilever. We demonstrate that the latter method is in particular useful for noncontact atomic force microscopy (NC-AFM) where the required simple instrumentation for spectral analysis is available in most experimental systems.

## Introduction

Noise as a result of thermal fluctuations is a ubiquitous phenomenon present in any physical system kept at a finite temperature. The seminal work of Nyquist established the simple framework of thermodynamic considerations for a quantitative description of such noise for a resistor kept at a temperature *T* and connected to an electrical network, as an example of a dynamic system in equilibrium with a thermal bath [1]. At the same time, it was pointed out by Johnson that such understanding is of great practical relevance as it allows for an optimisation of critical electronic devices with respect to their noise figures [2]. The main conclusion from this work is that a thermal bath provides a source of excitation with a strength that is constant over the entire frequency range, while the strength and spectral characteristics of the system response depends solely on the system transfer function. According to the equipartition theorem, the energy transferred from the thermal bath to a dynamic system equals (1/2)*k*_B_*T* for each degree of freedom, where *k*_B_ is the Boltzmann constant.

A cantilever is a mechanical dynamic system that is often described as a simple harmonic oscillator with a response function dominated by resonances at the eigenfrequencies *f_n_* of the flexural cantilever oscillation modes. Each of these modes represents a mechanical degree of freedom extracting (1/2)*k*_B_*T* of energy if connected to a thermal bath. The corresponding response to thermal excitation, namely the resulting noise power spectral density of the cantilever displacement 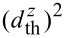, is the superposition of contributions from all modes and can be derived within the framework of the Nyquist theory [3]. Provided the simple harmonic oscillator model is valid, i.e., the internal damping of the cantilever is small, 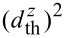 is given by

[1]
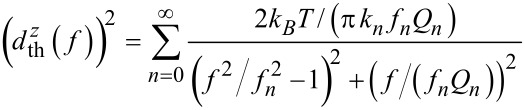


where *k_n_* and *Q_n_* are the modal stiffness [4] and *Q*-factor of the *n*th cantilever eigenmode [5], respectively. The relation is of relevance for any practical application involving microcantilevers and specifically important for high-resolution noncontact atomic force microscopy (NC-AFM) based on cantilever or tuning fork force sensors. We recently demonstrated how Equation 1 defines the ultimate limit of signal detection for an NC-AFM measurement performed under ultrahigh vacuum (UHV) conditions [6]. Although most NC-AFM systems are operated with cantilevers excited near their fundamental eigenfrequency *f*_0_, higher eigenmodes [7] have been investigated in the context of noise analysis [8], and it has been debated whether the thermal noise limitations in NC-AFM measurements could be reduced by operating cantilevers at higher eigenmodes [9]. It has further been realised that the cantilever properties *f_n_*, *k_n_* and *Q_n_* appear as linearly independent parameters in Equation 1. This allows their independent determination from a single measurement of the displacement noise spectral density 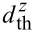 over a limited spectral range around the resonance for a cantilever kept at a known temperature [10]. A practical implementation of this notion, focused on the determination of cantilever stiffness from thermal noise, demonstrated the validity of the approach by a critical comparison of the results against corresponding results from other methods [11].

While the properties *f_n_* and *Q_n_* can quite easily be determined with high precision by a cantilever excitation experiment [12], the thermal method discussed here is hitherto the only one to yield valid results for the modal stiffness *k_n_*. The strength of the thermal method is that it is solely based on the equipartition theorem, establishing the simple energy balance [6]

[2]
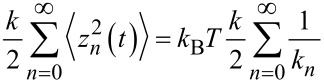


with *k* being the static stiffness of the cantilever.

This implies that a precise measurement of the mean square displacement 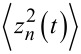 or the corresponding power spectral density (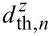
(*f*))^2^ in a region around a specific resonance *n* allows the determination of the modal stiffness *k_n_* without the knowledge of any other cantilever parameters such as dimensions, shape, mass or mass distribution.

Here, we critically discuss the extraction of intrinsic cantilever properties from measurements of thermal noise and focus on the precise determination of the modal stiffness *k_n_* as this quantity is a prerequisite for the quantitative interpretation of force imaging and spectroscopy results [13–15]. Most examples are given for the fundamental mode of the cantilever oscillation, but we also demonstrate that the method is universal and can equally well be applied to higher oscillation modes. The acquisition of noise spectra is, however, not trivial in this context as intrinsic *Q*-factors of the fundamental mode of high-*Q* cantilevers may be as high as 200,000. Thus, the accurate spectral analysis of the extremely narrow resonance peak requires expensive test equipment. Therefore, we introduce an alternative method of determining the modal stiffness by using the demodulator of an NC-AFM system to project the noise power of an excited cantilever around its resonance into the frequency range of 10 Hz to 1 kHz. Processing the resulting frequency shift signal Δ*f*(*t*) to obtain the modal stiffness in this frequency range is straightforward as a spectral analysis can be performed with simple equipment available in most NC-AFM control systems.

## Experimental

Measurements are performed in two UHV systems with NC-AFM instruments based on the optical beam deflection configuration. These have been described in our previous work as system B (UHV VT AFM/STM, Omicron NanoTechnology GmbH, Taunusstein, Germany) and as system C (UHV 750 variable temperature STM/AFM, RHK Technology, Inc., Troy, MI, USA) [6]. Temperatures used for data analysis are measured directly at the NC-AFM stage of a thermally equilibrated experimental system. It is, therefore, expected that the measured temperature is identical to the cantilever temperature. As test objects, we use a selection of four cantilever types with commercial names FM, NCH, Arrow™ and NCVH (Nanoworld AG, Neuchâtel, Switzerland). These cantilevers are chosen to cover a broad range of eigenfrequencies *f*_0_ ranging from 50 kHz to 2 MHz, static stiffness *k* [16] ranging from 3 to 120 N/m, and *Q*-factors *Q*_0_ [12] covering the range of 20,000 to 120,000; details are provided in Table 1. Measurements of the total displacement noise spectral density 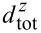
(*f*) are performed by using a spectrum analyser connected to the output of the preamplifier of the position-sensitive detector of the NC-AFM instrument. The cantilever displacement is measured as a calibrated electrical signal *V_z_*(*t*) and processed by the spectrum analyser [6]. For measurements of the total noise spectral density 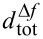
(*f_m_*) in the frequency shift signal Δ*f*(*t*), the spectrum analyser is connected to the phase-locked-loop (PLL) demodulator output of the respective NC-AFM system. In all of these experiments, utmost care has to be taken to shield the NC-AFM system from mechanical and, specifically, from electric noise in spectral regions encompassing the cantilever eigenfrequencies. Otherwise measurements may be severely false due to nonthermal noise contributions. Furthermore, valid results using this methodology can only be expected for thermal noise-limited measurements performed with a system for which the PLL transfer function is known. The former condition requires the detection system noise floor 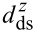 to be so low that, at least over a significant fraction of the PLL demodulator bandwidth, the frequency shift noise spectral density 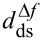
(*f_m_*) of the detection system is negligible compared to the thermal frequency-shift noise spectral density 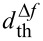
(*f_m_*) [6].

## Results and Discussion

### Stiffness from displacement thermal noise

In a displacement noise measurement of a cantilever with a high *Q*-factor, the spectrum analyser measures the total displacement noise spectral density 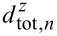
(*f*) for the *n*th cantilever oscillation mode, which can be represented as [6]

[3]
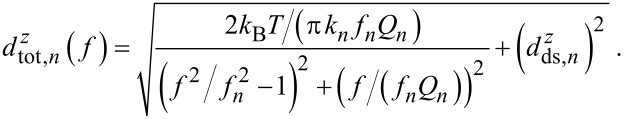


**Table 1:** Synopsis of cantilever properties. Cantilever dimensions are the length *l* (±2.5 µm), mean width 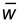
(±1.5 µm) and thickness *t* (±0.2 µm) as provided by the manufacturer. The stiffness *k*_dim_ is calculated from the cantilever dimensions, while *k*_stat_ is determined by a precision measurement of the static stiffness [16]. Eigenfrequency 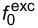 (standard deviation below 1 ppm) and quality factor 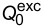 (standard deviation below 1%) are obtained from a fit of the simple harmonic oscillator transfer function to the measured resonance curve of the excited cantilever [12]. 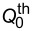 and 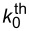 are the properties yielded when fitting Equation 3 to the displacement noise spectral density 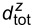 of a thermally excited cantilever. The value 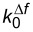 is extracted from the frequency shift noise 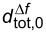 from Equation 4 by using 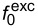 and 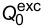 as known parameters.

cant.	*l* (µm)	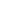 (µm)	*t* (µm)	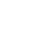 (Hz)	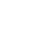	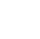	*k*_dim_ (N/m)	*k*_stat_ (N/m)	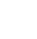 (N/m)	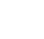 (N/m)

P 5	224	30	3.0	68,319	97,500	105,300	3.0 ± 0.9	2.73 ± 0.14	2.9	3.4
D 5	229	30	2.9	68,353	118,000	123,000	2.5 ± 0.8	2.50 ± 0.13	2.7	2.9
V 4	125	26	3.8	283,620	28,600	28,400	31 ± 6	25.2 ± 1.3	22	21
V 15	125	26	3.7	279,451	47,200	46,300	29 ± 6	—	24.3	22
AF 11	125	34	4.1	311,476	37,700	—	50 ± 13	44.6 ± 2.3	—	61
AL 3	—	—	—	1,316,757	16,600	—	9 ± 3^a^	—	—	8.7
AP 5	40	24	2.0	1,996,199	32,400	—	130 ± 50	—	—	125

^a^Value provided by the manufacturer.

**Figure 1:**
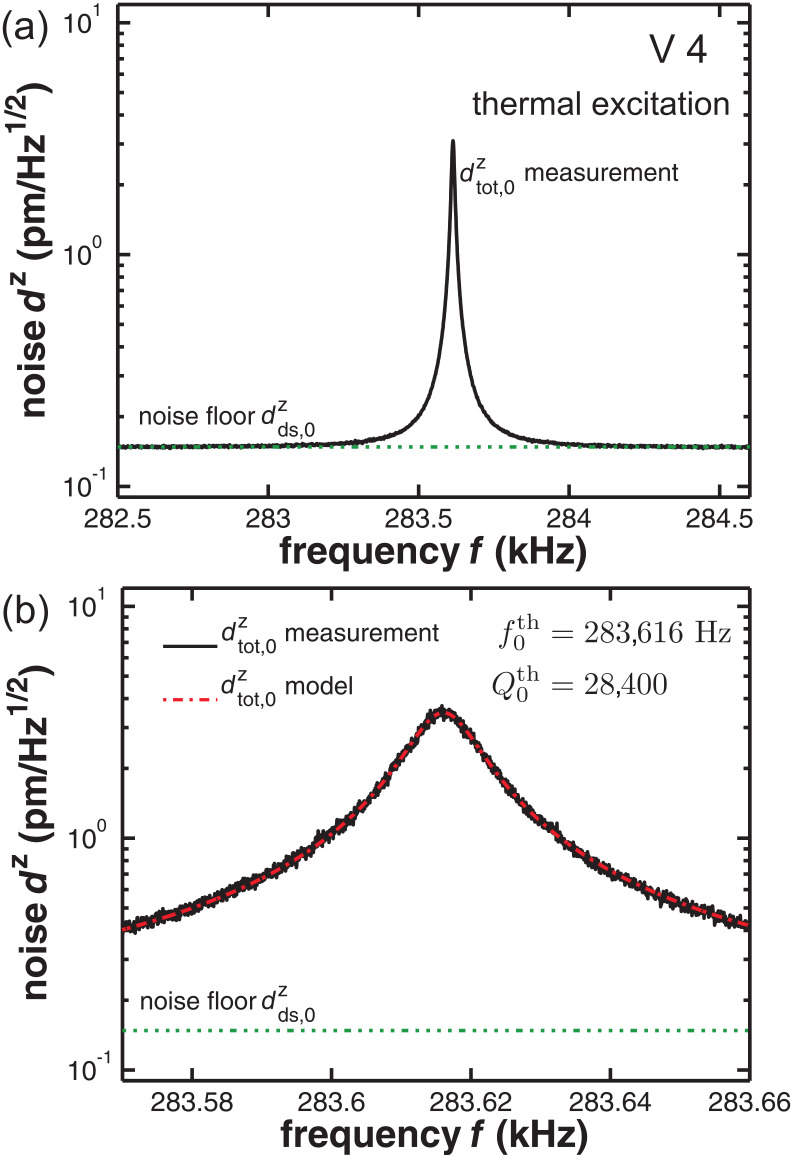
Displacement noise spectral density 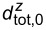 measured for the fundamental mode of cantilever V 4. Measurements represent the average of 50,000 spectra. (a) Measurement yielding the detection-system noise floor 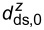 (dotted line). (b) Determination of 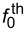, 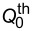 and 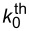 by a fit of Equation 3 to the measured spectrum (dash-dotted line).

**Figure 2:**
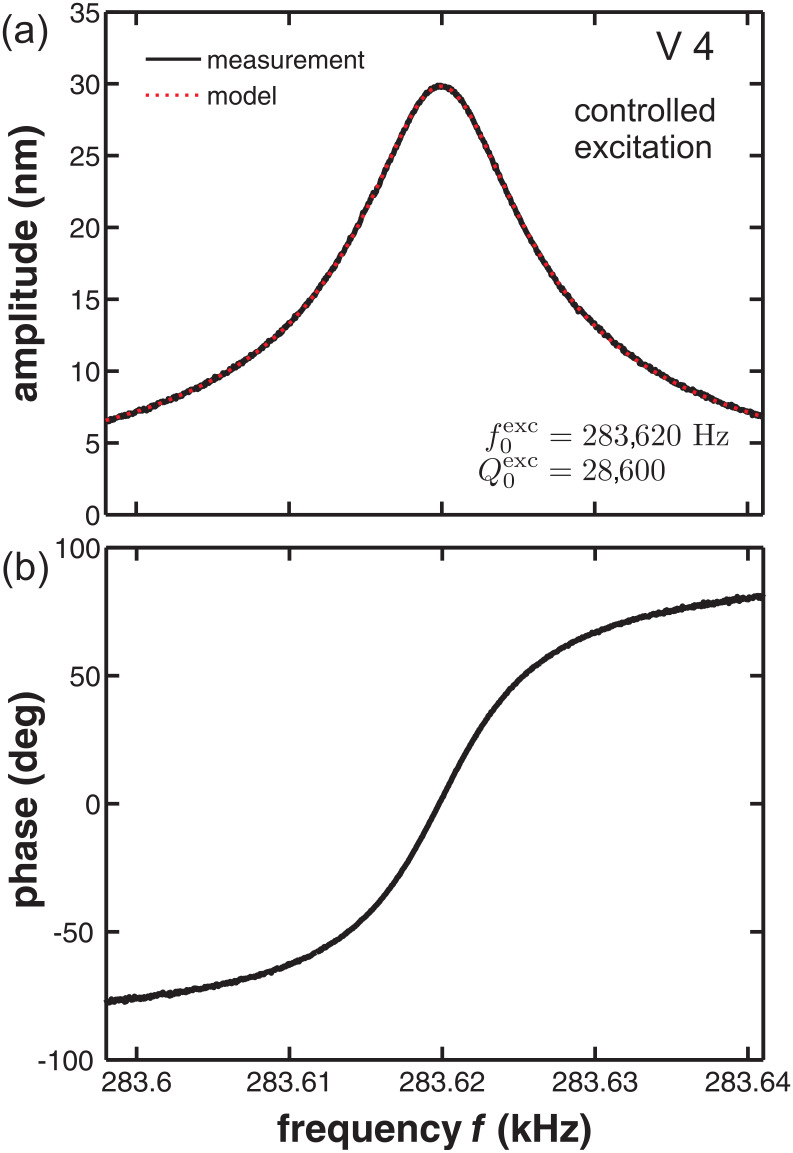
(a) Measured resonance curve (solid line) of the excited cantilever V 4 with a fit (dotted line) of Equation 3 from [12] to experimental data yielding 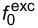 and 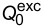. (b) Phase response of the excited cantilever V 4.

An exemplary spectrum of cantilever V 4 (see Table 1) covering the frequency region around the fundamental resonance at *f*_0_ ≈ 284 kHz is shown in Figure 1. The cantilever properties are extracted from the displacement noise spectrum by applying Equation 3. In the first step, the essentially white detection-system noise floor 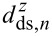 of the *n*th mode is determined by averaging the spectral density off resonance (see Figure 1a). In the second step, Equation 3 is fitted to the data with the cantilever properties *f*_0_, 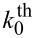 and 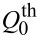 as fitting parameters and 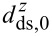 as determined in the first step (see Figure 1b). Respective measurements have been performed for many cantilevers with some results compiled in Table 1, together with information on cantilever dimensions and properties measured by other techniques. Thermal noise analysis of cantilever V 4, for instance, yields 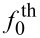 = 283,616 Hz, 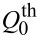 = 28,400 and 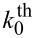 = 22 N/m. As a consistency check, we measure the cantilever response to excitation in the vicinity of the resonance, where the corresponding results for the amplitude and phase response are shown in Figure 2. A fit of the simple harmonic oscillator model to the amplitude response (Equation 3 in [12]) yields 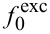 = 283,620 Hz and 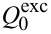 = 28,600 in excellent agreement with the thermal noise analysis. Generally, the fit of the thermal noise model from Equation 3 to the measured thermal excitation displacement data is excellent. In terms of experimental uncertainties, the highest precision is obtained in determining the eigenfrequency. State-of-the-art test equipment provides an accuracy of absolute frequency measurements below 1 ppm. However, practically the reproducibility is limited by thermal drift of the cantilever resonance between repeated measurements. This explains, for instance, the 4 Hz difference in the results for 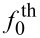 and 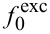 for cantilever V 4 as these measurements were performed with a time difference of several hours. The reproducibility in determining the *Q*-factor is determined by statistical errors and can be reduced to a standard deviation of 10% for 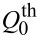 by appropriate averaging. Great care has to be taken, however, in mounting the cantilever to ensure that the measured *Q*-factor is the intrinsic *Q*-factor of the cantilever rather than an effective *Q*-factor caused by improper mounting of the cantilever [12]. Determining the cantilever stiffness 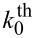 relies on an absolute measurement of the cantilever displacement. The main limitations of precision here are the uncertainty in the calibration of the cantilever oscillation amplitude [6,17–18] and of the electronic test equipment involved. The reproducibility for the measurement of 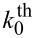 is typically 5% (standard deviation), while a comparison of 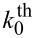 to values *k*_stat_ from reference measurements [16] yields an error of about 10% for the determination of stiffness from thermal noise. Note, however, that the modal stiffness *k*_0_ is related to the static stiffness *k* by *k*_0_ = 1.03*k* for a tipless cantilever while, for instance, a tip mass of 10% of the cantilever beam mass yields a relation of *k*_0_ = 1.01*k* instead [19].

In summary, the analysis of the displacement-noise spectral density around resonances of a thermally excited cantilever in a UHV environment allows the extraction of intrinsic cantilever properties with high accuracy and is specifically useful for determining modal stiffness. However, such measurements require a spectral analysis with high frequency resolution.

### Stiffness from frequency shift thermal noise

To circumvent the use of a high-resolution spectrum analyser and to facilitate measurements with the test equipment that is often integrated in NC-AFM control systems, we introduce an alternative method of extracting the cantilever modal stiffness from thermal noise. To apply this method, the eigenfrequency *f_n_* and the quality factor *Q_n_* of the *n*th oscillation mode have to be measured from an excited resonance curve as shown in Figure 2. To determine *k_n_*, the cantilever is excited to oscillation in the *n*th mode at a well stabilised amplitude *A_n_*. Thermal fluctuations described by the power spectral density given by Equation 1 are now superimposed to the deliberate oscillation. These fluctuations are detected by the PLL demodulator tuned to the cantilever eigenfrequency. Effectively, the PLL projects the displacement noise power spectral density in the sidebands of the mode resonance into a range of frequencies *f_m_* starting at 0 Hz. Considering the transfer function of the demodulation and the transfer function of the PLL output or loop filter *G*_filter_, the frequency shift noise spectral density at the PLL output can be represented as [6]

[5]
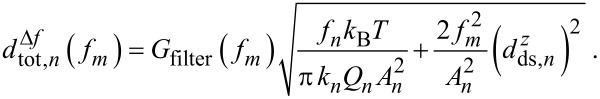


This allows us to obtain the modal stiffness from a measurement of 
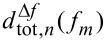
 if all other parameters are known:

[6]
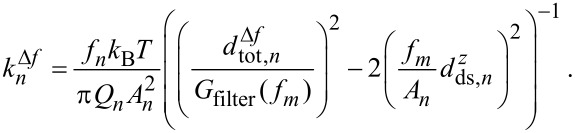


**Figure 3:**
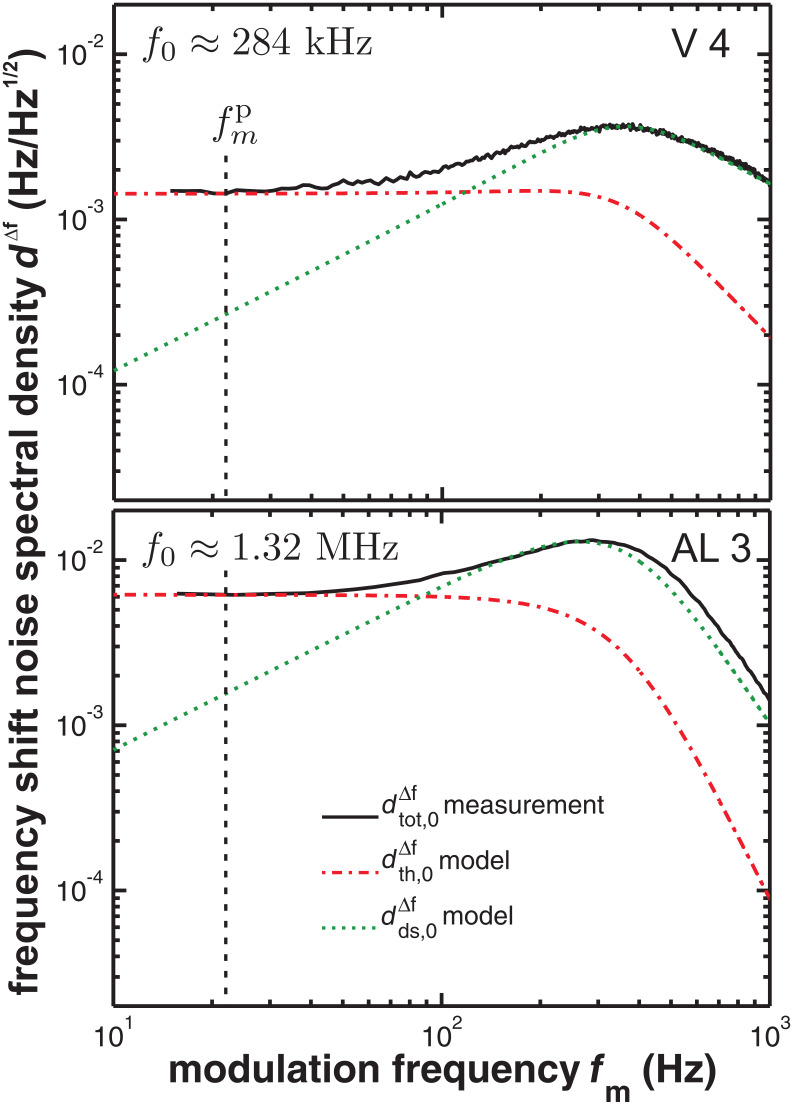
Frequency shift noise spectral density 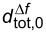 measured for cantilever V 4 (*A*_0_ = 16.8 nm, demodulator bandwidth *B*_−3dB_ = 415 Hz) and cantilever AL 3 (*A*_0_ = 16.5 nm, demodulator bandwidth *B*_−3dB_ = 258 Hz). Spectra are recorded with at least 1000 averages. Dotted lines show the contribution 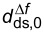 of the detection system noise floor; dash-dotted lines represent the contribution 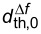 of the thermal noise to the total frequency shift noise 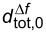. The analysis of the noise spectral density at the plateau frequency 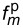 yields the cantilever stiffness 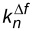 according to Equation 4.

Practically, the spectral analysis can be restricted to the frequency range of 10 Hz to 1 kHz. The resulting spectra are depicted in Figure 3 for different cantilevers (namely V 4 and AL 3 as described in Table 1) excited at their fundamental resonance mode at *f*_0_. The typical shape common to all such spectra has been explained in detail elsewhere [6]. The dotted and dash-dotted lines shown in Figure 3 represent the contributions from thermal noise and detection system frequency shift noise 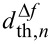 and 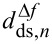, respectively. Here, the model curve for 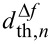 is not based on an independent measurement of 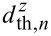, but determined from the measured 
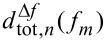
 curve assuming that the plateau indeed represents purely thermal noise. We focus on the plateau in 
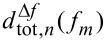
 found in the 10 to 50 Hz region. In the plateau region labelled by a representative modulation frequency 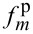, the frequency shift noise is dominated by thermal noise 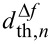
(*f_m_*) (dash-dotted line), while the noise contribution from the detection system 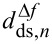
(*f_m_*) (dotted line) is negligible. Within this approximation, Equation 6 can be simplified to the following expression for the modal stiffness:

[4]
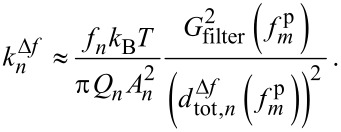


Prerequisite for a reliable determination of the modal stiffness is a knowledge of the cantilever properties *f_n_* and *Q_n_* and the PLL filter function *G*_filter_. While the former parameters can precisely be determined from an in situ cantilever excitation experiment [12], the latter function can be assumed to be 1 if the filter transfer function is reasonably well-behaved as a function of frequency and a sufficiently large PLL bandwidth is chosen [6]. Results shown in Figure 3 demonstrate that the signal quality obtained under typical experimental conditions is high enough to extract a well-defined value for 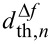
(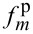) from the noise data. The modal stiffness values 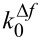 obtained for seven cantilevers for fundamental mode excitation according to Equation 4 are displayed in Table 1 and compared to the stiffness results obtained by using different methods for the same cantilevers. From these and further measurements (not shown here), we find an experimental uncertainty of about 20% for the modal stiffness obtained from the frequency shift noise. We attribute the decreased accuracy to noise and uncertainty in the calibration of the additional equipment involved. Note, however, that any noise source besides the thermal excitation yields a reduction in the measured modal stiffness and cannot explain values that are too high.

The determination of the effective cantilever stiffness from frequency shift noise is most interesting for cantilever excitation at higher oscillation modes where the projection of the displacement noise into the low frequency region by the PLL demodulator is especially convenient. Respective results obtained for cantilevers P 5 and AF 11 are shown in Table 2. For cantilever P 5 we obtain *k*_1_ = 154 N/m and *k*_2_ = 1330 N/m. As there is no reference for a cross-check of these values, we check for plausibility within the framework of the cantilever oscillation theory. Equation 6 given in [4] allows us to calculate the modal stiffness for a given ratio of tip mass to beam mass. Assuming the tip mass being 2% of the cantilever beam mass yields *k*_1_/*k*_0_ = 45.0 and *k*_2_/*k*_0_ = 397. These numbers fit well to the measured values for cantilever P 5 (see Table 2). The result for AF 11 can be explained by a tip mass of 5% of the cantilever beam mass.

**Table 2:** Cantilever eigenfrequencies 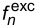 and quality factors 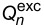 of the *n*th oscillating mode for cantilevers P 5 and AF 11. The modal stiffness 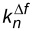 is obtained from the frequency shift noise spectral density 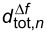 through Equation 4. 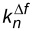/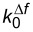 is the ratio of the modal stiffness of the *n*th oscillation mode to the stiffness of the fundamental mode.

cantilever	*n*	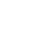 (Hz)	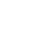	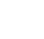 (N/m)	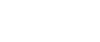

P 5	1	436,711	44,900	154	45.3
P 5	2	1,234,277	5841	1330	391
AF 11	1	1,934,677	9000	3420	56

## Conclusion

In conclusion, we introduce a method for determining the modal stiffness *k_n_* of a cantilever from frequency shift noise complemented by an independent measurement of the modal eigenfrequency and *Q*-factor. Our strategy yields valid results with an uncertainty of about 20%; however, the accuracy is expected to be increased by an improvement of the experimental setup. This method is particularly convenient as measurements can be performed with simple test equipment implemented in many NC-AFM control systems. Additionally, the involved spectral analysis is simple and can be performed over a bandwidth of only 1 kHz at maximum, irrespective of the cantilever eigenfrequency. We apply the thermal noise method to various cantilever types and find a good agreement of these cantilever parameters with those determined by using alternative methods. The strength of the methods presented here is that they directly yield the modal stiffness derived from a thermal measurement and do not require any modelling to relate geometric cantilever properties to oscillation properties.
